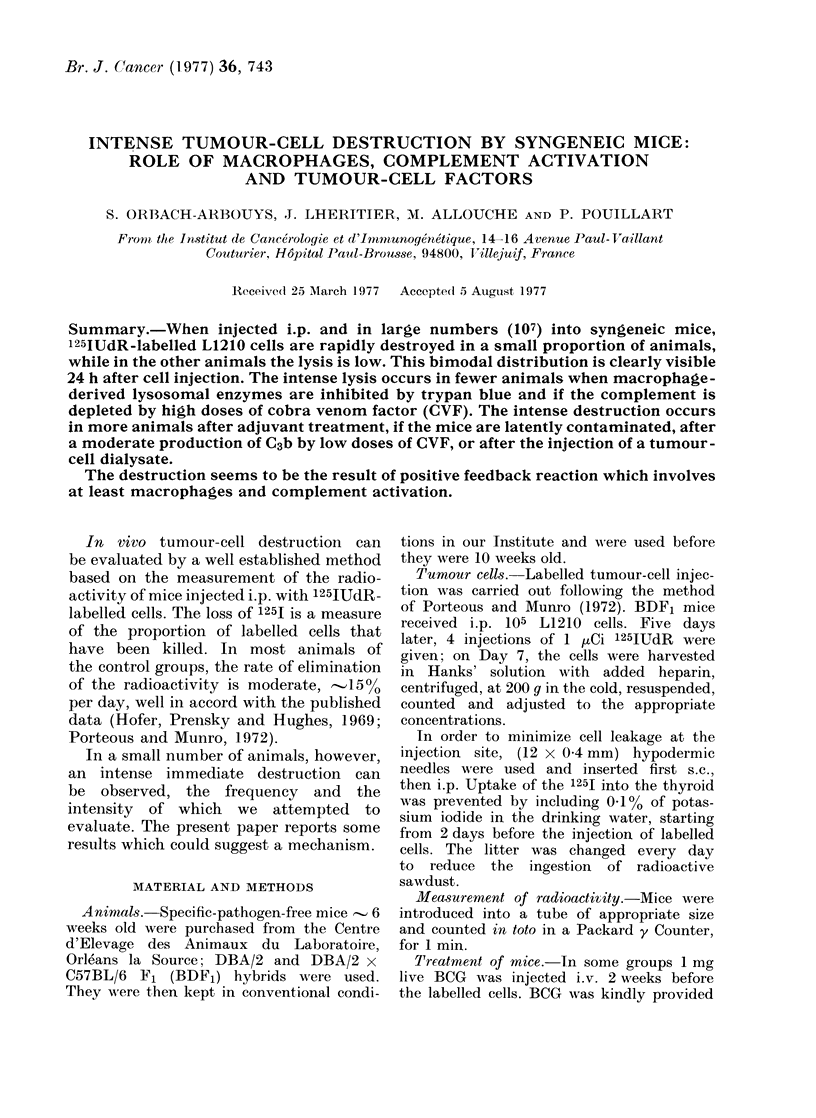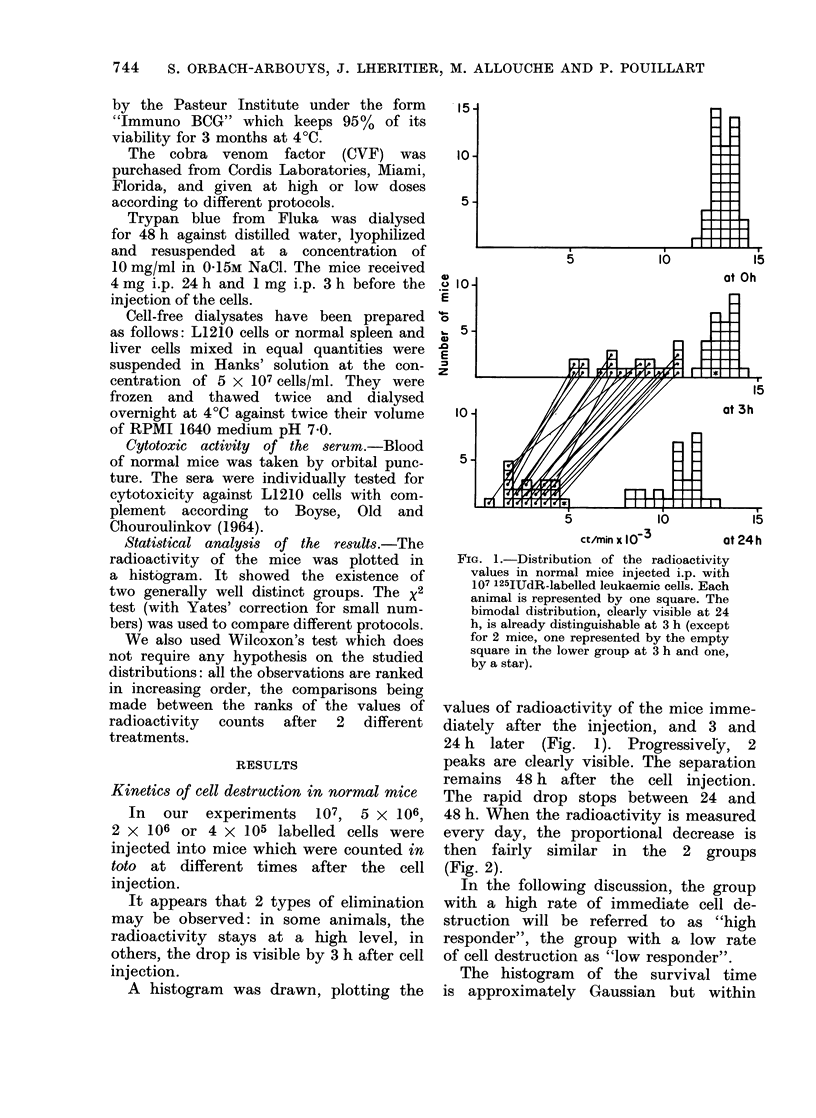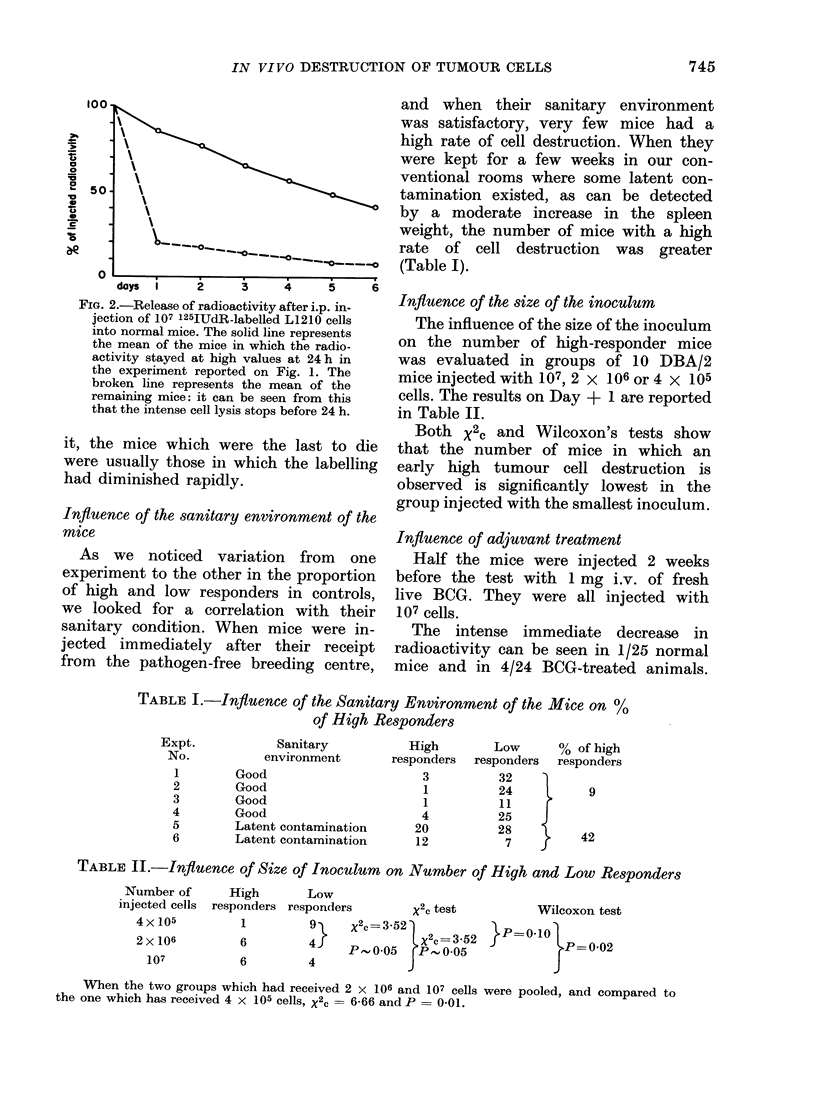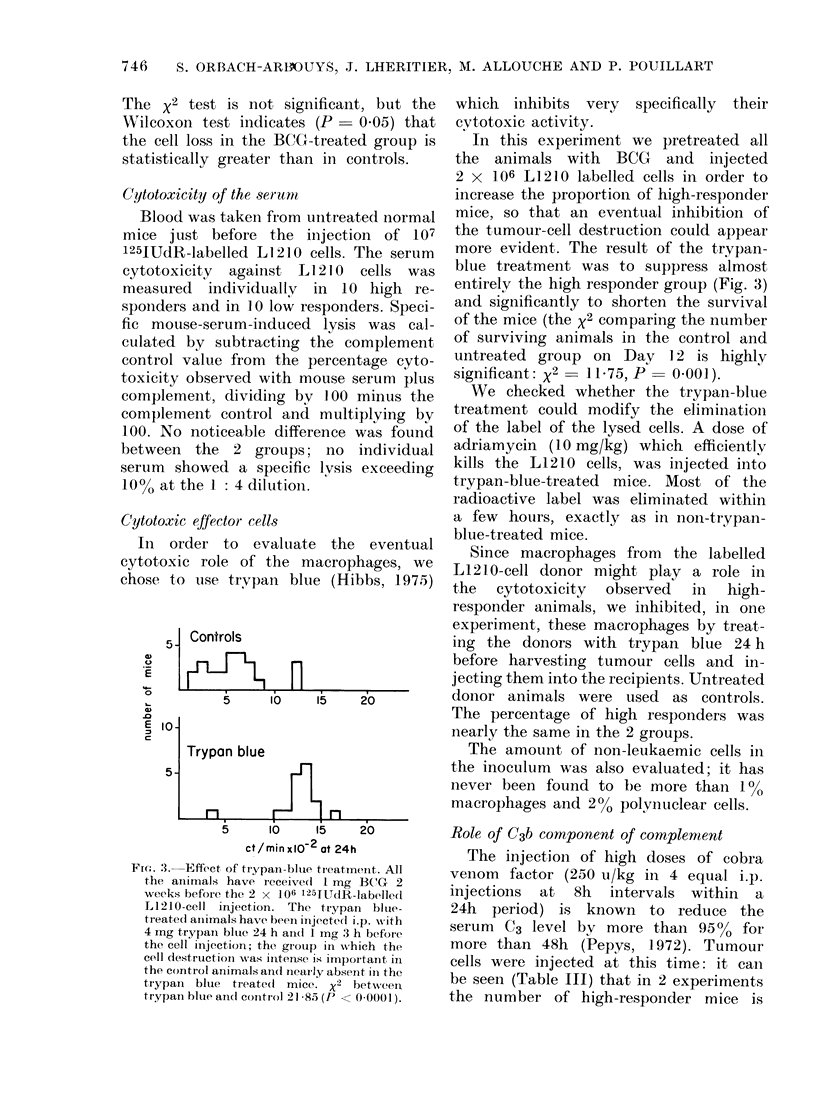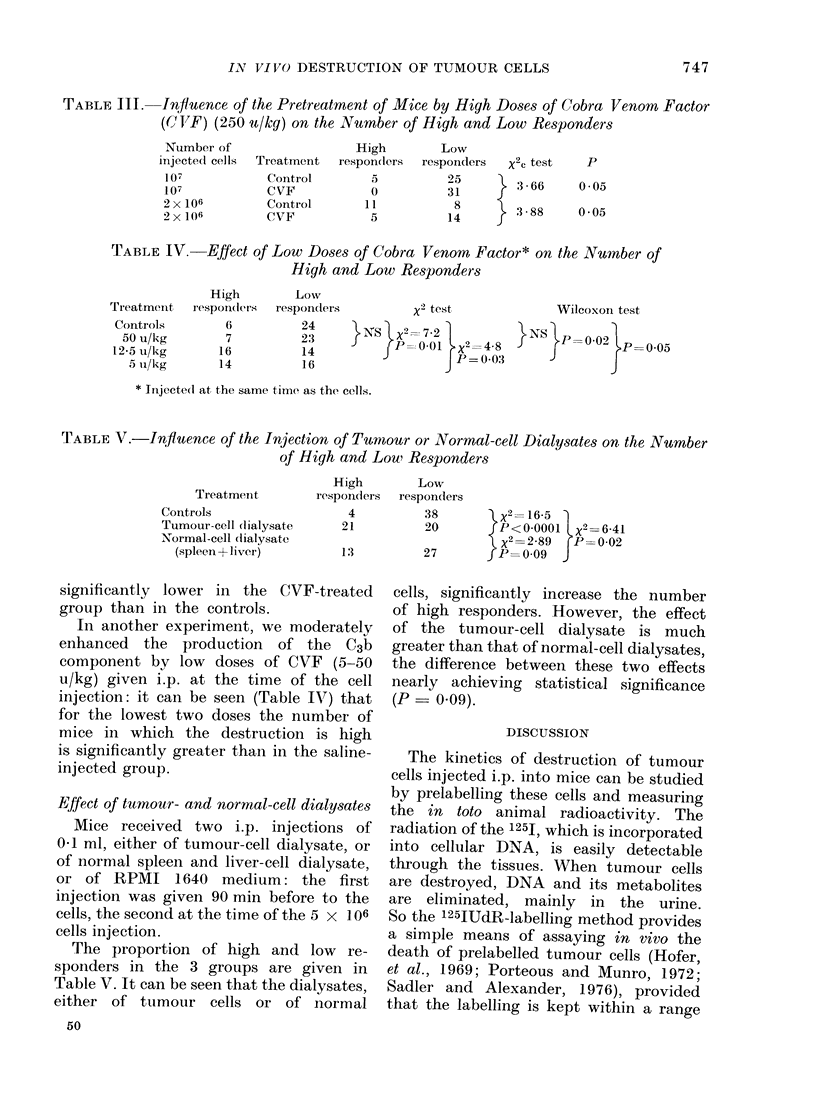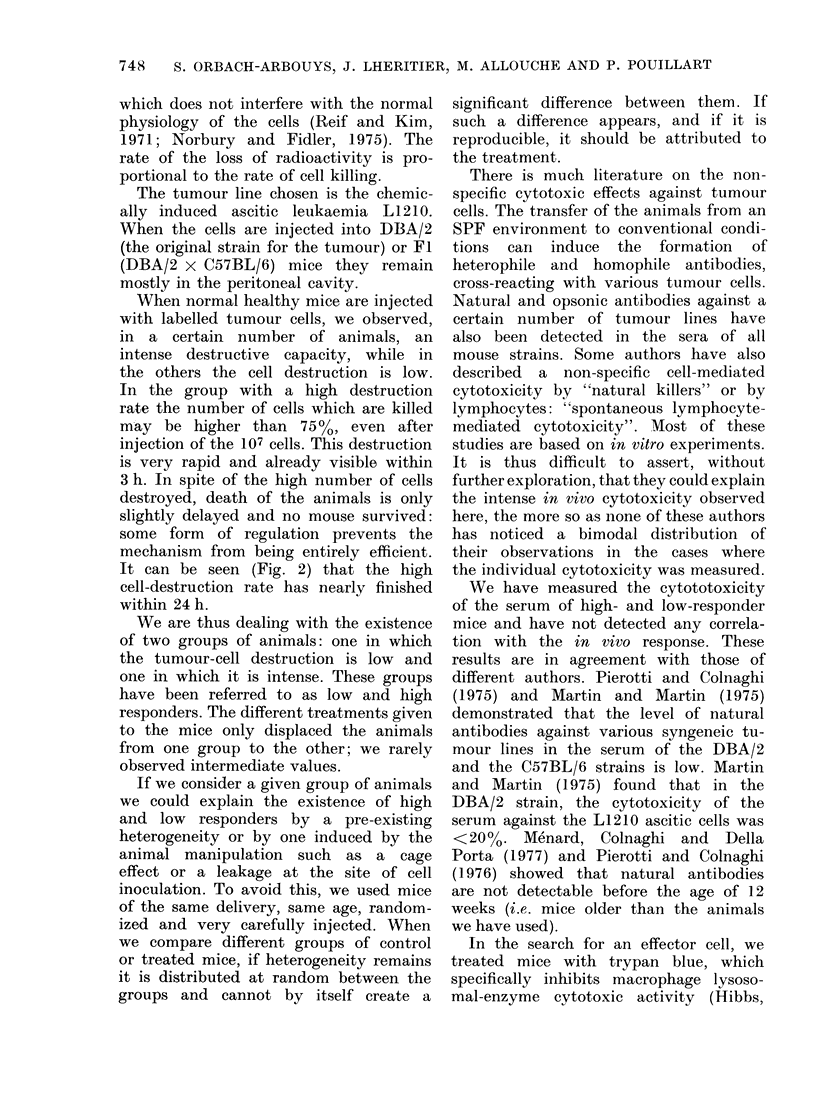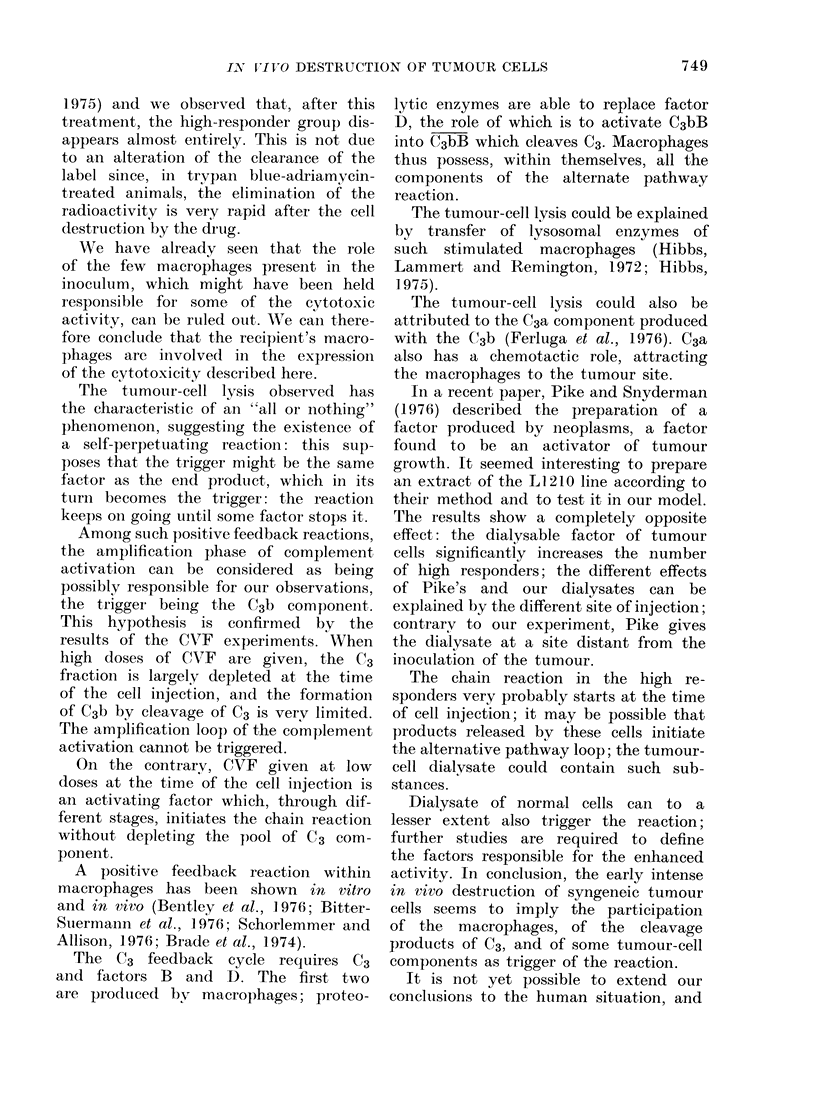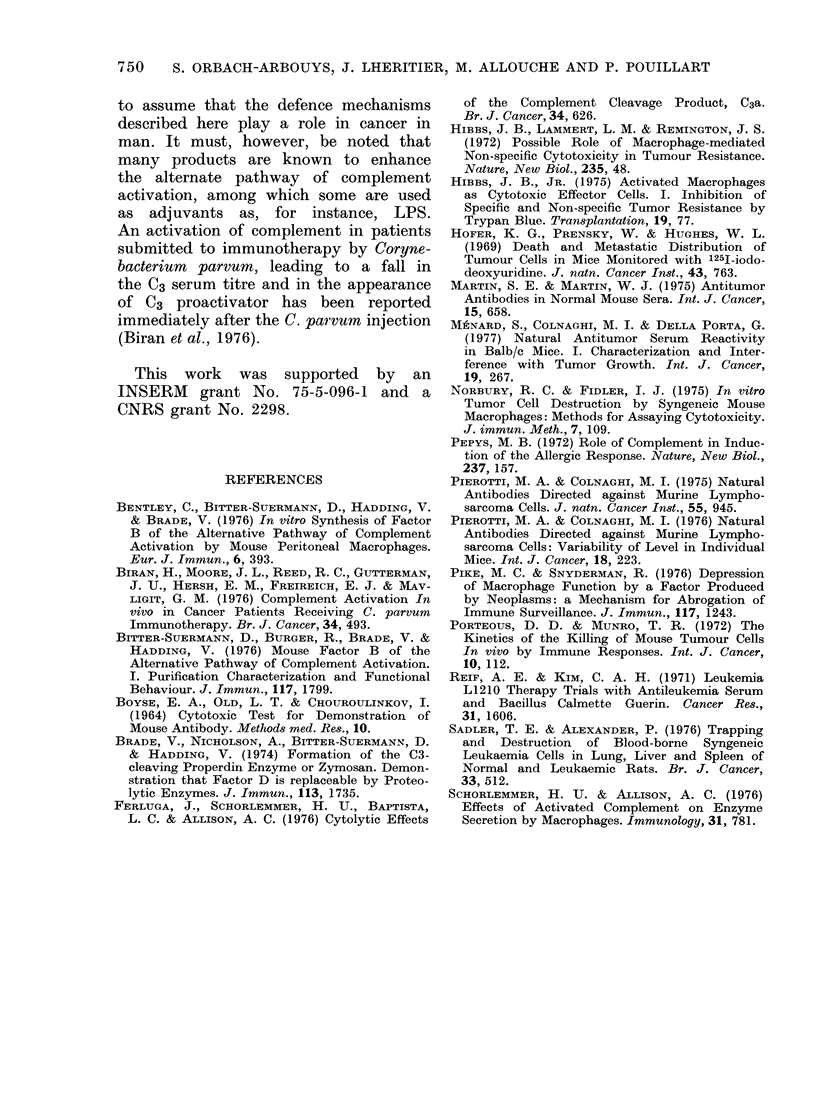# Intense tumour-cell destruction by syngeneic mice: role of macrophages, complement activation and tumour-cell factors.

**DOI:** 10.1038/bjc.1977.257

**Published:** 1977-12

**Authors:** S. Orbach-Arbouys, J. Lheritier, M. Allouche, P. Pouillart

## Abstract

When injected i.p. and in large numbers (10(7)) into syngeneic mice, 125IUdR-labelled L1210 cells are rapidly destroyed in a small proportion of animals, while in the other animals the lysis is low. This bimodal distribution is clearly visible 24 h after cell injection. The intense lysis occurs in fewer animals when macrophage-derived lysosomal enzymes are inhibited by trypan blue and if the complement is depleted by high doses of cobra venom factor (CVF). The intense destruction occurs in more animals after adjuvant treatment, if the mice are latently contaminated, after a moderate production of C3b by low doses of CVF, or after the injection of a tumour-cell dialysate. The destruction seems to be the result of positive feedback reaction which involves at least macrophages and complement activation.


*Br. J. Cancer* (1977) **36**, 743

### INTENSE TUMOUR-CELL DESTRUCTION BY SYNGENEIC MICE: ROLE OF MACROPHAGES, COMPLEMENT ACTIVATION AND TUMOUR-CELL FACTORS

S. ORBACH-ARBOUYS, J. LHERITIER, M. ALLOUCHE AND P. POUILLART

*From the Institut de Cancérologie et d'Immunogénétique, 14–16 Avenue Paul-Vaillant Couturier, Hôpital Paul-Brousse, 94800, Villejuif, France*

Received 25 March 1977 Accepted 5 August 1977

**Summary.—When injected i.p. and in large numbers ($10^7$) into syngeneic mice, $^{125}$IUdR-labelled L1210 cells are rapidly destroyed in a small proportion of animals, while in the other animals the lysis is low. This bimodal distribution is clearly visible 24 h after cell injection. The intense lysis occurs in fewer animals when macrophage-derived lysosomal enzymes are inhibited by trypan blue and if the complement is depleted by high doses of cobra venom factor (CVF). The intense destruction occurs in more animals after adjuvant treatment, if the mice are latently contaminated, after a moderate production of C3b by low doses of CVF, or after the injection of a tumour-cell dialysate.**

**The destruction seems to be the result of positive feedback reaction which involves at least macrophages and complement activation.**

*In vivo* tumour-cell destruction can be evaluated by a well established method based on the measurement of the radioactivity of mice injected i.p. with $^{125}$IUdR-labelled cells. The loss of $^{125}$I is a measure of the proportion of labelled cells that have been killed. In most animals of the control groups, the rate of elimination of the radioactivity is moderate, ~15% per day, well in accord with the published data (Hofer, Prensky and Hughes, 1969; Porteous and Munro, 1972).

In a small number of animals, however, an intense immediate destruction can be observed, the frequency and the intensity of which we attempted to evaluate. The present paper reports some results which could suggest a mechanism.

### MATERIAL AND METHODS

*Animals.*—Specific-pathogen-free mice ~ 6 weeks old were purchased from the Centre d'Elevage des Animaux du Laboratoire, Orléans la Source; DBA/2 and DBA/2 × C57BL/6 $F_1$ ($BDF_1$) hybrids were used. They were then kept in conventional conditions in our Institute and were used before they were 10 weeks old.

*Tumour cells.*—Labelled tumour-cell injection was carried out following the method of Porteous and Munro (1972). $BDF_1$ mice received i.p. $10^5$ L1210 cells. Five days later, 4 injections of 1 $\mu$Ci $^{125}$IUdR were given; on Day 7, the cells were harvested in Hanks' solution with added heparin, centrifuged, at 200 *g* in the cold, resuspended, counted and adjusted to the appropriate concentrations.

In order to minimize cell leakage at the injection site, (12 × 0·4 mm) hypodermic needles were used and inserted first s.c., then i.p. Uptake of the $^{125}$I into the thyroid was prevented by including 0·1% of potassium iodide in the drinking water, starting from 2 days before the injection of labelled cells. The litter was changed every day to reduce the ingestion of radioactive sawdust.

*Measurement of radioactivity.*—Mice were introduced into a tube of appropriate size and counted *in toto* in a Packard $\gamma$ Counter, for 1 min.

*Treatment of mice.*—In some groups 1 mg live BCG was injected i.v. 2 weeks before the labelled cells. BCG was kindly provided by the Pasteur Institute under the form "Immuno BCG" which keeps 95% of its viability for 3 months at 4°C.

The cobra venom factor (CVF) was purchased from Cordis Laboratories, Miami, Florida, and given at high or low doses according to different protocols.

Trypan blue from Fluka was dialysed for 48 h against distilled water, lyophilized and resuspended at a concentration of 10 mg/ml in 0·15M NaCl. The mice received 4 mg i.p. 24 h and 1 mg i.p. 3 h before the injection of the cells.

Cell-free dialysates have been prepared as follows: L1210 cells or normal spleen and liver cells mixed in equal quantities were suspended in Hanks' solution at the concentration of $5 \times 10^7$ cells/ml. They were frozen and thawed twice and dialysed overnight at 4°C against twice their volume of RPMI 1640 medium pH 7·0.

*Cytotoxic activity of the serum.*—Blood of normal mice was taken by orbital puncture. The sera were individually tested for cytotoxicity against L1210 cells with complement according to Boyse, Old and Chouroulinkov (1964).

*Statistical analysis of the results.*—The radioactivity of the mice was plotted in a histogram. It showed the existence of two generally well distinct groups. The $\chi^2$ test (with Yates' correction for small numbers) was used to compare different protocols.

We also used Wilcoxon's test which does not require any hypothesis on the studied distributions: all the observations are ranked in increasing order, the comparisons being made between the ranks of the values of radioactivity counts after 2 different treatments.

### RESULTS

### *Kinetics of cell destruction in normal mice*

In our experiments $10^7$, $5 \times 10^6$, $2 \times 10^6$ or $4 \times 10^5$ labelled cells were injected into mice which were counted *in toto* at different times after the cell injection.

It appears that 2 types of elimination may be observed: in some animals, the radioactivity stays at a high level, in others, the drop is visible by 3 h after cell injection.

A histogram was drawn, plotting the values of radioactivity of the mice immediately after the injection, and 3 and 24 h later (Fig. 1). Progressively, 2 peaks are clearly visible. The separation remains 48 h after the cell injection. The rapid drop stops between 24 and 48 h. When the radioactivity is measured every day, the proportional decrease is then fairly similar in the 2 groups (Fig. 2).

FIG. 1.—Distribution of the radioactivity values in normal mice injected i.p. with $10^7$ $^{125}$IUdR-labelled leukaemic cells. Each animal is represented by one square. The bimodal distribution, clearly visible at 24 h, is already distinguishable at 3 h (except for 2 mice, one represented by the empty square in the lower group at 3 h and one, by a star).

In the following discussion, the group with a high rate of immediate cell destruction will be referred to as "high responder", the group with a low rate of cell destruction as "low responder".

The histogram of the survival time is approximately Gaussian but within

FIG. 2.—Release of radioactivity after i.p. injection of $10^7$ $^{125}$IUdR-labelled L1210 cells into normal mice. The solid line represents the mean of the mice in which the radioactivity stayed at high values at 24 h in the experiment reported on Fig. 1. The broken line represents the mean of the remaining mice: it can be seen from this that the intense cell lysis stops before 24 h.

it, the mice which were the last to die were usually those in which the labelling had diminished rapidly.

*Influence of the sanitary environment of the mice*

As we noticed variation from one experiment to the other in the proportion of high and low responders in controls, we looked for a correlation with their sanitary condition. When mice were injected immediately after their receipt from the pathogen-free breeding centre, and when their sanitary environment was satisfactory, very few mice had a high rate of cell destruction. When they were kept for a few weeks in our conventional rooms where some latent contamination existed, as can be detected by a moderate increase in the spleen weight, the number of mice with a high rate of cell destruction was greater (Table I).

*Influence of the size of the inoculum*

The influence of the size of the inoculum on the number of high-responder mice was evaluated in groups of 10 DBA/2 mice injected with $10^7$, $2 \times 10^6$ or $4 \times 10^5$ cells. The results on Day + 1 are reported in Table II.

Both $\chi^2_c$ and Wilcoxon's tests show that the number of mice in which an early high tumour cell destruction is observed is significantly lowest in the group injected with the smallest inoculum.

*Influence of adjuvant treatment*

Half the mice were injected 2 weeks before the test with 1 mg i.v. of fresh live BCG. They were all injected with $10^7$ cells.

The intense immediate decrease in radioactivity can be seen in 1/25 normal mice and in 4/24 BCG-treated animals.

TABLE I.—*Influence of the Sanitary Environment of the Mice on % of High Responders*

| Expt. No. | Sanitary environment | High responders | Low responders | % of high responders |
|---|---|---|---|---|
| 1 | Good | 3 | 32 | 9 (Expts 1–4) |
| 2 | Good | 1 | 24 | |
| 3 | Good | 1 | 11 | |
| 4 | Good | 4 | 25 | |
| 5 | Latent contamination | 20 | 28 | 42 (Expts 5–6) |
| 6 | Latent contamination | 12 | 7 | |

TABLE II.—*Influence of Size of Inoculum on Number of High and Low Responders*

| Number of injected cells | High responders | Low responders | $\chi^2_c$ test | Wilcoxon test |
|---|---|---|---|---|
| $4 \times 10^5$ | 1 | 9 | $\chi^2_c = 3{\cdot}52$, $P \sim 0{\cdot}05$ ($4\times10^5$ vs $2\times10^6$); $\chi^2_c = 3{\cdot}52$, $P \sim 0{\cdot}05$ ($2\times10^6$ vs $10^7$) | $P = 0{\cdot}10$ ($4\times10^5$ vs $2\times10^6$); $P = 0{\cdot}02$ (all groups) |
| $2 \times 10^6$ | 6 | 4 | | |
| $10^7$ | 6 | 4 | | |

When the two groups which had received $2 \times 10^6$ and $10^7$ cells were pooled, and compared to the one which has received $4 \times 10^5$ cells, $\chi^2_c = 6{\cdot}66$ and $P = 0{\cdot}01$.

The $\chi^2$ test is not significant, but the Wilcoxon test indicates ($P = 0{\cdot}05$) that the cell loss in the BCG-treated group is statistically greater than in controls.

*Cytotoxicity of the serum*

Blood was taken from untreated normal mice just before the injection of $10^7$ $^{125}$IUdR-labelled L1210 cells. The serum cytotoxicity against L1210 cells was measured individually in 10 high responders and in 10 low responders. Specific mouse-serum-induced lysis was calculated by subtracting the complement control value from the percentage cytotoxicity observed with mouse serum plus complement, dividing by 100 minus the complement control and multiplying by 100. No noticeable difference was found between the 2 groups; no individual serum showed a specific lysis exceeding 10% at the 1 : 4 dilution.

*Cytotoxic effector cells*

In order to evaluate the eventual cytotoxic role of the macrophages, we chose to use trypan blue (Hibbs, 1975)

FIG. 3.—Effect of trypan-blue treatment. All the animals have received 1 mg BCG 2 weeks before the 2 × $10^6$ $^{125}$IUdR-labelled L1210-cell injection. The trypan blue-treated animals have been injected i.p. with 4 mg trypan blue 24 h and 1 mg 3 h before the cell injection; the group in which the cell destruction was intense is important in the control animals and nearly absent in the trypan blue treated mice. $\chi^2$ between trypan blue and control 21·85 ($P < 0{\cdot}0001$).

which inhibits very specifically their cytotoxic activity.

In this experiment we pretreated all the animals with BCG and injected 2 × $10^6$ L1210 labelled cells in order to increase the proportion of high-responder mice, so that an eventual inhibition of the tumour-cell destruction could appear more evident. The result of the trypan-blue treatment was to suppress almost entirely the high responder group (Fig. 3) and significantly to shorten the survival of the mice (the $\chi^2$ comparing the number of surviving animals in the control and untreated group on Day 12 is highly significant: $\chi^2 = 11{\cdot}75$, $P = 0{\cdot}001$).

We checked whether the trypan-blue treatment could modify the elimination of the label of the lysed cells. A dose of adriamycin (10 mg/kg) which efficiently kills the L1210 cells, was injected into trypan-blue-treated mice. Most of the radioactive label was eliminated within a few hours, exactly as in non-trypan-blue-treated mice.

Since macrophages from the labelled L1210-cell donor might play a role in the cytotoxicity observed in high-responder animals, we inhibited, in one experiment, these macrophages by treating the donors with trypan blue 24 h before harvesting tumour cells and injecting them into the recipients. Untreated donor animals were used as controls. The percentage of high responders was nearly the same in the 2 groups.

The amount of non-leukaemic cells in the inoculum was also evaluated; it has never been found to be more than 1% macrophages and 2% polynuclear cells.

*Role of $C_3b$ component of complement*

The injection of high doses of cobra venom factor (250 u/kg in 4 equal i.p. injections at 8h intervals within a 24h period) is known to reduce the serum $C_3$ level by more than 95% for more than 48h (Pepys, 1972). Tumour cells were injected at this time: it can be seen (Table III) that in 2 experiments the number of high-responder mice is

TABLE III.—*Influence of the Pretreatment of Mice by High Doses of Cobra Venom Factor (CVF) (250 u/kg) on the Number of High and Low Responders*

| Number of injected cells | Treatment | High responders | Low responders | $\chi^2_c$ test | $P$ |
|---|---|---|---|---|---|
| $10^7$ | Control | 5 | 25 | 3·66 | 0·05 |
| $10^7$ | CVF | 0 | 31 | | |
| $2 \times 10^6$ | Control | 11 | 8 | 3·88 | 0·05 |
| $2 \times 10^6$ | CVF | 5 | 14 | | |

TABLE IV.—*Effect of Low Doses of Cobra Venom Factor* on the Number of High and Low Responders*

| Treatment | High responders | Low responders | $\chi^2$ test | | | Wilcoxon test | | |
|---|---|---|---|---|---|---|---|---|
| Controls | 6 | 24 | NS | $\chi^2 = 7{\cdot}2$ | | NS | $P = 0{\cdot}02$ | |
| 50 u/kg | 7 | 23 | | $P = 0{\cdot}01$ | $\chi^2 = 4{\cdot}8$ | | | $P = 0{\cdot}05$ |
| 12·5 u/kg | 16 | 14 | | | $P = 0{\cdot}03$ | | | |
| 5 u/kg | 14 | 16 | | | | | | |

* Injected at the same time as the cells.

TABLE V.—*Influence of the Injection of Tumour or Normal-cell Dialysates on the Number of High and Low Responders*

| Treatment | High responders | Low responders | | |
|---|---|---|---|---|
| Controls | 4 | 38 | $\chi^2 = 16{\cdot}5$ | |
| Tumour-cell dialysate | 21 | 20 | $P < 0{\cdot}0001$ | $\chi^2 = 6{\cdot}41$ |
| Normal-cell dialysate (spleen + liver) | 13 | 27 | $\chi^2 = 2{\cdot}89$, $P = 0{\cdot}09$ | $P = 0{\cdot}02$ |

significantly lower in the CVF-treated group than in the controls.

In another experiment, we moderately enhanced the production of the C3b component by low doses of CVF (5–50 u/kg) given i.p. at the time of the cell injection: it can be seen (Table IV) that for the lowest two doses the number of mice in which the destruction is high is significantly greater than in the saline-injected group.

### *Effect of tumour- and normal-cell dialysates*

Mice received two i.p. injections of 0·1 ml, either of tumour-cell dialysate, or of normal spleen and liver-cell dialysate, or of RPMI 1640 medium: the first injection was given 90 min before to the cells, the second at the time of the $5 \times 10^6$ cells injection.

The proportion of high and low responders in the 3 groups are given in Table V. It can be seen that the dialysates, either of tumour cells or of normal cells, significantly increase the number of high responders. However, the effect of the tumour-cell dialysate is much greater than that of normal-cell dialysates, the difference between these two effects nearly achieving statistical significance ($P = 0{\cdot}09$).

### DISCUSSION

The kinetics of destruction of tumour cells injected i.p. into mice can be studied by prelabelling these cells and measuring the *in toto* animal radioactivity. The radiation of the $^{125}$I, which is incorporated into cellular DNA, is easily detectable through the tissues. When tumour cells are destroyed, DNA and its metabolites are eliminated, mainly in the urine. So the $^{125}$IUdR-labelling method provides a simple means of assaying *in vivo* the death of prelabelled tumour cells (Hofer, *et al.*, 1969; Porteous and Munro, 1972; Sadler and Alexander, 1976), provided that the labelling is kept within a range which does not interfere with the normal physiology of the cells (Reif and Kim, 1971; Norbury and Fidler, 1975). The rate of the loss of radioactivity is proportional to the rate of cell killing.

The tumour line chosen is the chemically induced ascitic leukaemia L1210. When the cells are injected into DBA/2 (the original strain for the tumour) or F1 (DBA/2 × C57BL/6) mice they remain mostly in the peritoneal cavity.

When normal healthy mice are injected with labelled tumour cells, we observed, in a certain number of animals, an intense destructive capacity, while in the others the cell destruction is low. In the group with a high destruction rate the number of cells which are killed may be higher than 75%, even after injection of the $10^7$ cells. This destruction is very rapid and already visible within 3 h. In spite of the high number of cells destroyed, death of the animals is only slightly delayed and no mouse survived: some form of regulation prevents the mechanism from being entirely efficient. It can be seen (Fig. 2) that the high cell-destruction rate has nearly finished within 24 h.

We are thus dealing with the existence of two groups of animals: one in which the tumour-cell destruction is low and one in which it is intense. These groups have been referred to as low and high responders. The different treatments given to the mice only displaced the animals from one group to the other; we rarely observed intermediate values.

If we consider a given group of animals we could explain the existence of high and low responders by a pre-existing heterogeneity or by one induced by the animal manipulation such as a cage effect or a leakage at the site of cell inoculation. To avoid this, we used mice of the same delivery, same age, randomized and very carefully injected. When we compare different groups of control or treated mice, if heterogeneity remains it is distributed at random between the groups and cannot by itself create a significant difference between them. If such a difference appears, and if it is reproducible, it should be attributed to the treatment.

There is much literature on the non-specific cytotoxic effects against tumour cells. The transfer of the animals from an SPF environment to conventional conditions can induce the formation of heterophile and homophile antibodies, cross-reacting with various tumour cells. Natural and opsonic antibodies against a certain number of tumour lines have also been detected in the sera of all mouse strains. Some authors have also described a non-specific cell-mediated cytotoxicity by "natural killers" or by lymphocytes: "spontaneous lymphocyte-mediated cytotoxicity". Most of these studies are based on *in vitro* experiments. It is thus difficult to assert, without further exploration, that they could explain the intense *in vivo* cytotoxicity observed here, the more so as none of these authors has noticed a bimodal distribution of their observations in the cases where the individual cytotoxicity was measured.

We have measured the cytototoxicity of the serum of high- and low-responder mice and have not detected any correlation with the *in vivo* response. These results are in agreement with those of different authors. Pierotti and Colnaghi (1975) and Martin and Martin (1975) demonstrated that the level of natural antibodies against various syngeneic tumour lines in the serum of the DBA/2 and the C57BL/6 strains is low. Martin and Martin (1975) found that in the DBA/2 strain, the cytotoxicity of the serum against the L1210 ascitic cells was <20%. Ménard, Colnaghi and Della Porta (1977) and Pierotti and Colnaghi (1976) showed that natural antibodies are not detectable before the age of 12 weeks (*i.e.* mice older than the animals we have used).

In the search for an effector cell, we treated mice with trypan blue, which specifically inhibits macrophage lysosomal-enzyme cytotoxic activity (Hibbs,

1975) and we observed that, after this treatment, the high-responder group disappears almost entirely. This is not due to an alteration of the clearance of the label since, in trypan blue-adriamycin-treated animals, the elimination of the radioactivity is very rapid after the cell destruction by the drug.

We have already seen that the role of the few macrophages present in the inoculum, which might have been held responsible for some of the cytotoxic activity, can be ruled out. We can therefore conclude that the recipient's macrophages are involved in the expression of the cytotoxicity described here.

The tumour-cell lysis observed has the characteristic of an "all or nothing" phenomenon, suggesting the existence of a self-perpetuating reaction: this supposes that the trigger might be the same factor as the end product, which in its turn becomes the trigger: the reaction keeps on going until some factor stops it.

Among such positive feedback reactions, the amplification phase of complement activation can be considered as being possibly responsible for our observations, the trigger being the $C_3b$ component. This hypothesis is confirmed by the results of the CVF experiments. When high doses of CVF are given, the $C_3$ fraction is largely depleted at the time of the cell injection, and the formation of $C_3b$ by cleavage of $C_3$ is very limited. The amplification loop of the complement activation cannot be triggered.

On the contrary, CVF given at low doses at the time of the cell injection is an activating factor which, through different stages, initiates the chain reaction without depleting the pool of $C_3$ component.

A positive feedback reaction within macrophages has been shown *in vitro* and *in vivo* (Bentley *et al.*, 1976; Bitter-Suermann *et al.*, 1976; Schorlemmer and Allison, 1976; Brade *et al.*, 1974).

The $C_3$ feedback cycle requires $C_3$ and factors B and D. The first two are produced by macrophages; proteolytic enzymes are able to replace factor D, the role of which is to activate $C_3bB$ into $\overline{C_3bB}$ which cleaves $C_3$. Macrophages thus possess, within themselves, all the components of the alternate pathway reaction.

The tumour-cell lysis could be explained by transfer of lysosomal enzymes of such stimulated macrophages (Hibbs, Lammert and Remington, 1972; Hibbs, 1975).

The tumour-cell lysis could also be attributed to the $C_3a$ component produced with the $C_3b$ (Ferluga *et al.*, 1976). $C_3a$ also has a chemotactic role, attracting the macrophages to the tumour site.

In a recent paper, Pike and Snyderman (1976) described the preparation of a factor produced by neoplasms, a factor found to be an activator of tumour growth. It seemed interesting to prepare an extract of the L1210 line according to their method and to test it in our model. The results show a completely opposite effect: the dialysable factor of tumour cells significantly increases the number of high responders; the different effects of Pike's and our dialysates can be explained by the different site of injection; contrary to our experiment, Pike gives the dialysate at a site distant from the inoculation of the tumour.

The chain reaction in the high responders very probably starts at the time of cell injection; it may be possible that products released by these cells initiate the alternative pathway loop; the tumour-cell dialysate could contain such substances.

Dialysate of normal cells can to a lesser extent also trigger the reaction; further studies are required to define the factors responsible for the enhanced activity. In conclusion, the early intense *in vivo* destruction of syngeneic tumour cells seems to imply the participation of the macrophages, of the cleavage products of $C_3$, and of some tumour-cell components as trigger of the reaction.

It is not yet possible to extend our conclusions to the human situation, and to assume that the defence mechanisms described here play a role in cancer in man. It must, however, be noted that many products are known to enhance the alternate pathway of complement activation, among which some are used as adjuvants as, for instance, LPS. An activation of complement in patients submitted to immunotherapy by *Corynebacterium parvum*, leading to a fall in the $C_3$ serum titre and in the appearance of $C_3$ proactivator has been reported immediately after the *C. parvum* injection (Biran *et al.*, 1976).

This work was supported by an INSERM grant No. 75-5-096-1 and a CNRS grant No. 2298.

### REFERENCES

BENTLEY, C., BITTER-SUERMANN, D., HADDING, V. & BRADE, V. (1976) *In vitro* Synthesis of Factor B of the Alternative Pathway of Complement Activation by Mouse Peritoneal Macrophages. *Eur. J. Immun.*, **6**, 393.

BIRAN, H., MOORE, J. L., REED, R. C., GUTTERMAN, J. U., HERSH, E. M., FREIREICH, E. J. & MAVLIGIT, G. M. (1976) Complement Activation *In vivo* in Cancer Patients Receiving *C. parvum* Immunotherapy. *Br. J. Cancer*, **34**, 493.

BITTER-SUERMANN, D., BURGER, R., BRADE, V. & HADDING, V. (1976) Mouse Factor B of the Alternative Pathway of Complement Activation. I. Purification Characterization and Functional Behaviour. *J. Immun.*, **117**, 1799.

BOYSE, E. A., OLD, L. T. & CHOUROULINKOV, I. (1964) Cytotoxic Test for Demonstration of Mouse Antibody. *Methods med. Res.*, **10**.

BRADE, V., NICHOLSON, A., BITTER-SUERMANN, D. & HADDING, V. (1974) Formation of the C3-cleaving Properdin Enzyme or Zymosan. Demonstration that Factor D is replaceable by Proteolytic Enzymes. *J. Immun.*, **113**, 1735.

FERLUGA, J., SCHORLEMMER, H. U., BAPTISTA, L. C. & ALLISON, A. C. (1976) Cytolytic Effects of the Complement Cleavage Product, $C_3a$. *Br. J. Cancer*, **34**, 626.

HIBBS, J. B., LAMMERT, L. M. & REMINGTON, J. S. (1972) Possible Role of Macrophage-mediated Non-specific Cytotoxicity in Tumour Resistance. *Nature, New Biol.*, **235**, 48.

HIBBS, J. B., JR. (1975) Activated Macrophages as Cytotoxic Effector Cells. I. Inhibition of Specific and Non-specific Tumor Resistance by Trypan Blue. *Transplantation*, **19**, 77.

HOFER, K. G., PRENSKY, W. & HUGHES, W. L. (1969) Death and Metastatic Distribution of Tumour Cells in Mice Monitored with $^{125}$I-iododeoxyuridine. *J. natn. Cancer Inst.*, **43**, 763.

MARTIN, S. E. & MARTIN, W. J. (1975) Antitumor Antibodies in Normal Mouse Sera. *Int. J. Cancer*, **15**, 658.

MÉNARD, S., COLNAGHI, M. I. & DELLA PORTA, G. (1977) Natural Antitumor Serum Reactivity in Balb/c Mice. I. Characterization and Interference with Tumor Growth. *Int. J. Cancer*, **19**, 267.

NORBURY, R. C. & FIDLER, I. J. (1975) *In vitro* Tumor Cell Destruction by Syngeneic Mouse Macrophages: Methods for Assaying Cytotoxicity. *J. immun. Meth.*, **7**, 109.

PEPYS, M. B. (1972) Role of Complement in Induction of the Allergic Response. *Nature, New Biol.*, **237**, 157.

PIEROTTI, M. A. & COLNAGHI, M. I. (1975) Natural Antibodies Directed against Murine Lymphosarcoma Cells. *J. natn. Cancer Inst.*, **55**, 945.

PIEROTTI, M. A. & COLNAGHI, M. I. (1976) Natural Antibodies Directed against Murine Lymphosarcoma Cells: Variability of Level in Individual Mice. *Int. J. Cancer*, **18**, 223.

PIKE, M. C. & SNYDERMAN, R. (1976) Depression of Macrophage Function by a Factor Produced by Neoplasms: a Mechanism for Abrogation of Immune Surveillance. *J. Immun.*, **117**, 1243.

PORTEOUS, D. D. & MUNRO, T. R. (1972) The Kinetics of the Killing of Mouse Tumour Cells *In vivo* by Immune Responses. *Int. J. Cancer*, **10**, 112.

REIF, A. E. & KIM, C. A. H. (1971) Leukemia L1210 Therapy Trials with Antileukemia Serum and Bacillus Calmette Guerin. *Cancer Res.*, **31**, 1606.

SADLER, T. E. & ALEXANDER, P. (1976) Trapping and Destruction of Blood-borne Syngeneic Leukaemia Cells in Lung, Liver and Spleen of Normal and Leukaemic Rats. *Br. J. Cancer*, **33**, 512.

SCHORLEMMER, H. U. & ALLISON, A. C. (1976) Effects of Activated Complement on Enzyme Secretion by Macrophages. *Immunology*, **31**, 781.